# Utility of the dual-specificity protein kinase TTK as a therapeutic target for intrahepatic spread of liver cancer

**DOI:** 10.1038/srep33121

**Published:** 2016-09-13

**Authors:** Ruoyu Miao, Yan Wu, Haohai Zhang, Huandi Zhou, Xiaofeng Sun, Eva Csizmadia, Lian He, Yi Zhao, Chengyu Jiang, Rebecca A. Miksad, Tahereh Ghaziani, Simon C. Robson, Haitao Zhao

**Affiliations:** 1 Department of Liver Surgery, Peking Union Medical College Hospital, Chinese Academy of Medical Sciences and Peking Union Medical College, Beijing 100730, China; 2 Liver Center and The Transplant Institute, Department of Medicine, Beth Israel Deaconess Medical Center, Harvard Medical School, Boston, MA 02115, USA; 3 State Key Laboratory of Medical Molecular Biology, Institute of Basic Medical Sciences, Chinese Academy of Medical Sciences and Peking Union Medical College, Beijing 100005, China; 4 Key Lab of Intelligent Information Processing of Chinese Academy of Sciences, Institute of Computing Technology, Beijing 100190, China; 5 Division of Hematology/Oncology, Department of Medicine, Beth Israel Deaconess Medical Center, Harvard Medical School, Boston, MA 02115, USA

## Abstract

Therapies for primary liver cancer, the third leading cause of cancer-related death worldwide, remain limited. Following multi-omics analysis (including whole genome and transcriptome sequencing), we were able to identify the dual-specific protein kinase TTK as a putative new prognostic biomarker for liver cancer. Herein, we show that levels of TTK protein are significantly elevated in neoplastic tissues from a cohort of liver cancer patients, when compared with adjacent hepatic tissues. We also tested the utility of TTK targeted inhibition and have demonstrated therapeutic potential in an experimental model of liver cancer *in vivo*. Following lentiviral shRNA knockdown in several human liver cancer cell lines, we demonstrated that TTK boosts cell growth and promotes cell spreading; as well as protects against senescence and decreases autophagy. In an experimental animal model, we show that *in vitro* knockdown of TTK effectively blocks intrahepatic growth of human HCC xenografts. Furthermore, we note that, *in vivo* silencing of TTK, by systemically delivering TTK siRNAs to already tumor-bearing liver, limits intrahepatic spread of liver cancer cells. This intervention is associated with decreased tumor aggressiveness, as well as increased senescence and autophagy. Taken together, our data suggest that targeted TTK inhibition might have clinical utility as an adjunct therapy in management of liver cancer.

Hepatocellular carcinoma (HCC), the fifth most common malignant cancer globally, is the most common form of primary liver cancer and has a dismal prognosis^1^,^2^. HCC is the common malignant disease that develops in cirrhotic and fibrotic livers and is linked to a variety of etiologies: hepatitis B virus (HBV), hepatitis C virus (HCV), and/or toxic/metabolic liver injury, diabetes and obesity^3^,^4^. Although multiple treatment options are available, these provide limited benefits and are associated with substantive side effects. Standard approaches to therapy include surgical resection, liver transplantation, loco-regional treatments (e.g. radiofrequency ablation as well as various forms of transarterial embolization and radiation) and systemic therapy treatments with cytotoxic chemotherapy or, more recently, targeted therapy such as sorafenib, a multikinase inhibitor.

Treatment choices are often dictated by the extent and location of tumor burden, liver function and overall condition of the patient^3^,^5^. However, outcomes remain heterogeneous and assessment of biology, natural course and aggressiveness still represent major clinical challenges. Predicting treatment response and durability remains poorly characterized. The current clinically used biomarkers such as alpha-fetoprotein (AFP) fail to meet the needs for accurate prognosis and treatment stratification of HCC because of low sensitivity and specificity^6^,^7^.

A better biomarker would allow for better clinical stratification and may facilitate highly individualized and targeted treatment of liver cancer. Recently, using a multi-omics approach (inclusive of whole genome and transcriptome sequencing), we identified TTK, a dual-specificity protein kinase that was thought to be involved in mitotic spindle assembly checkpoint and control of cell cycle program, as a bona fide biomarker for liver cancer with prognostic significance in a large cohort of liver cancer patients (*n* = 174)^8^. We found that heightened intratumoral expression of TTK correlates with aggressive clinical course and low survival in liver cancer^8^. Independently, Liang *et al.* demonstrated that TTK is associated with acquired sorafenib-resistance in various liver cancer cell lines^9^. However, little is still known regarding the role of TTK in hepatocarcinogenesis.

We have put forward the hypothesis that TTK has pro-carcinogenic roles in HCC development and progression. We believe TTK is operational via augmentation of primary cancer cell growth and spread. Directed TTK blockade might serve as an effective therapeutic target. Therefore, we have evaluated the role of TTK in *in vitro* cell culture systems and developed studies of therapeutic utility in pre-clinical *in vivo* animal models.

## Results

### TTK protein levels are elevated in human liver cancer

**Figure 1:**
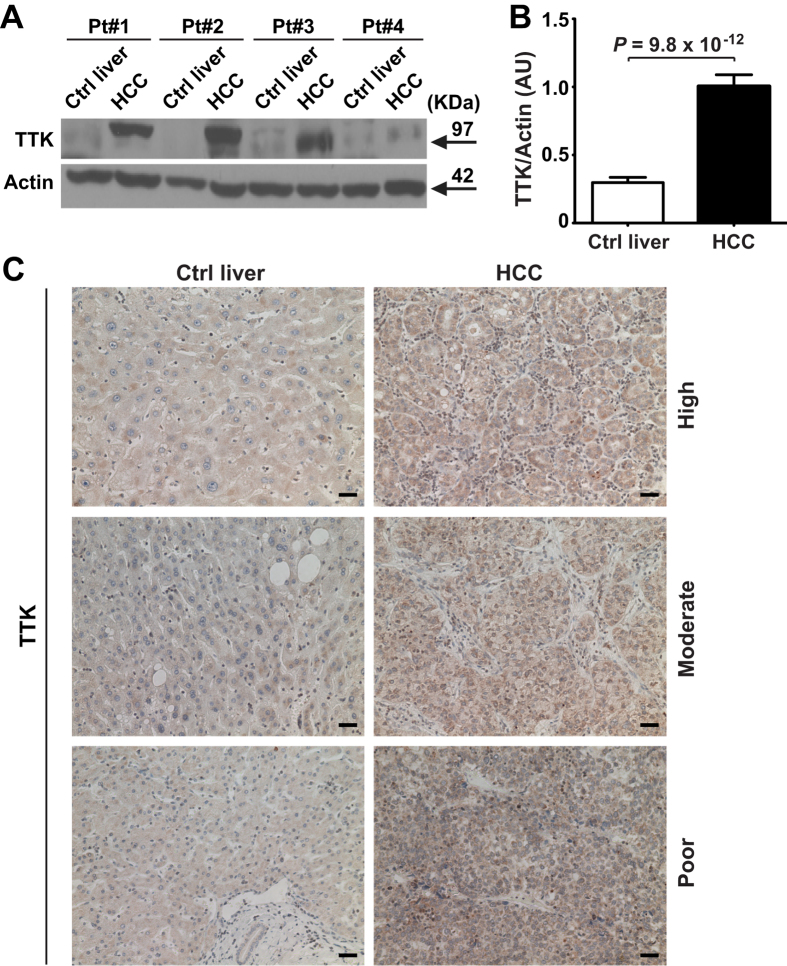
Protein levels of TTK are increased in human HCC, independently of histological differentiation grade. (**A,B**) Representative Western blot images of TTK (**A**) and densitometric analyses of the TTK protein bands (**B**) in HCC and adjacent HCC-free liver tissues (Ctrl liver) (*n* = 34). (**C**) Representative images of immunohistochemical staining of TTK in formalin-fixed, paraffin-embedded tissue specimens (*n* = 18). Grade of tumor differentiation is shown on the right. Scale bar, 20 μm. Error bars, mean ± s.e.m, paired Student’s *t*-test. AU, arbitrary units.

Western blot results showed that TTK protein levels recapitulated mRNA expression patterns in 34 pairs of HBV-HCC tumor and adjacent noncancerous liver tissues^8^: protein levels of TTK were significantly increased in liver cancer tissues, when compared to noncancerous liver tissues (*P* = 9.8 × 10^−12^) (Fig. 1A,B and Fig. S1). Indeed, 30 out of 34 cancer-free tissues were completely negative for TTK (Fig. S1). This observation was further validated by immunohistochemical staining using clinical samples from another independent patient cohort (*n* = 18). As shown in Fig. 1C and Fig. S2, regardless of the tumor differentiation grade, elevation of expressed TTK protein was clearly noted in cancer tissues.

Next, in order to circumvent TTK antibody-created non-specificity in both Western blot and immunohistochemical staining^10^, we preformed a preliminary RNAscope *in situ* hybridization (ISH) assay using freshly made formalin-fixed, paraffin-embedded tissue blocks from a HBV-HCC patient. We noted that tumor tissues had positive staining of TTK, whereas tumor-free liver tissues were totally or nearly absent of TTK (Fig. S3). In this RNA ISH assay, peptidylprolyl isomerase B (PPIB), a protein highly expressed in liver cancer served as a positive control and dihydrodipicolinate reductase (dapB) as the negative control.

These results are concordant with data in the public domain i.e. human tumor database Oncomine. Here, TTK levels are likewise significantly increased in human HCC tissue samples when compared to control liver tissues (www.Oncomine.org).

### TTK knockdown inhibits growth and malignant potential of human liver cancer cells *in vitro*

In order to evaluate the therapeutic potential of TTK against liver cancer, we first examined the impact of *in vitro* gene silencing of TTK on cellular function of liver cancer cells. TTK were expressed at both mRNA and protein levels in all human HCC cell lines used in this study, including HepG2, Huh7, Hep3B, PLC/PRL/5, and SK-HEP-1 (a HCC-associated endothelial cell line) (Fig. S4).

We next generated stable TTK deficient HCC cells using lentiviral shRNA methods. These cell lines displayed differential sensitivity to TTK inhibition. Specifically, we could only produce TTK deficiency in HepG2 and Huh7 cell lines, whereas knockdown of TTK was lethal for Hep3B and PLC/PRL/5 cells (data not shown).

**Figure 2:**
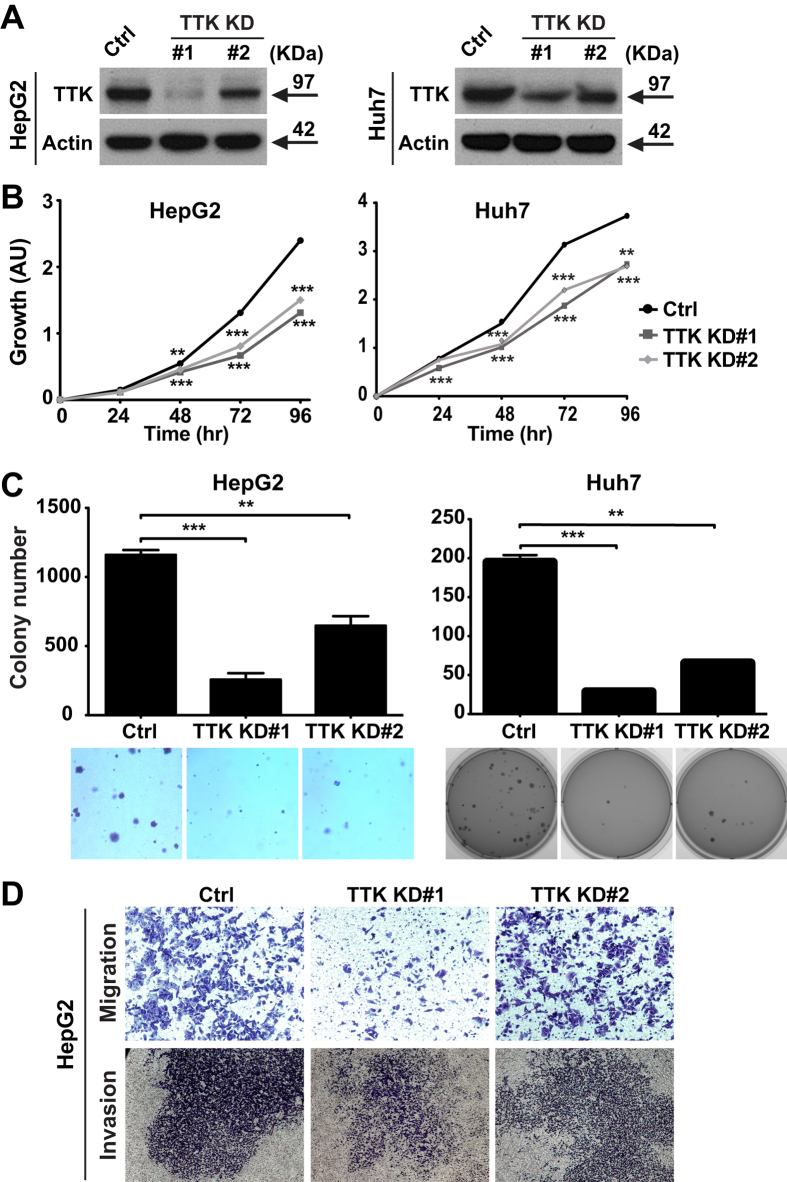
Knockdown of TTK limits the growth, migration and invasiveness of HCC cells *in vitro*. (**A**) Validation of TTK knockdown in HepG2 and Huh7 cells by Western blotting. Ctrl, empty shRNA vector control; TTK KD#1–2: two TTK-specific shRNAs. (**B**) Impacts of TTK knockdown on cell growth evaluated by CCK-8 that measures the activity of cellular dehydrogenases (correlating with cell viability). (**C**) Clonogenicity of TTK KD cells measured by soft agar 3D colony formation assay. Top, quantification of colonies; bottom, representative images of colonies. (**D**) Malignant potential of HepG2 TTK KD cells assessed by migration and invasion assays. *n* = 3–8; error bars, mean ± s.e.m, Student’s *t*-test. In contrast to control knockdown (Ctrl) cells, **P < 0.01; ***P < 0.0001. AU, arbitrary units.

As such, HepG2 and Huh7 cells were used for subsequent experimentation. In both cell lines, two out of four shRNAs specifically targeting human TTK, efficiently blocked TTK expression at both mRNA and protein levels, albeit with differential silencing potency (Fig. 2A and Fig. S5). Knockdown (KD) of TTK significantly decreased cell growth ability and “clonogenicity” of HCC cells, as compared with the control (Ctrl) counterparts (Fig. 2B,C and Fig. S6). Moreover, the malignant capacity of cells, a critical determinant of liver cancer recurrence after liver resection or liver transplantation, was assessed using both migration and invasion assays. Marked decreases in both migratory capacity and invasiveness were noted in TTK KD cells (Fig. 2D and Fig. S7).

### TTK deficiency induces cell death of liver cancer cells *in vitro*

**Figure 3:**
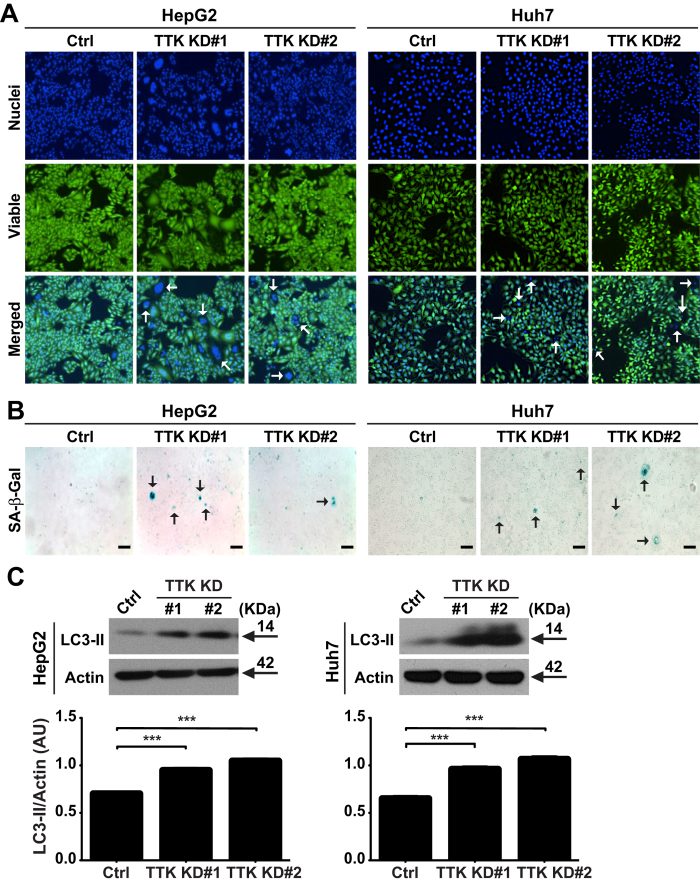
Relative TTK deficiency leads to enhanced senescent and autophagic cell death. (**A**) Representative images of live cells by Celigo *in situ* cellular analysis. Top, nuclei stained by Hoechst 33342; middle, viable cells stained by Calcein AM; bottom, merged images. Arrows indicate dying/dead cells. (**B**) Cellular senescence indicated by SA-β-Gal activity. Arrows indicate senescent cells. (**C**) Immunoblot (top) and densitometric analysis (bottom) of LC3-II, a sensitive indicator of cellular autophagy. Scale bar, 40 μm. *n* = 3; error bars, mean ± s.e.m Student’s *t*-test. ***P < 0.0001. AU, arbitrary units.

We investigated the functional consequences of TTK inhibition on liver cancer cell death, inclusive of apoptosis, senescence and autophagy. By performing Celigo *in situ* cellular analysis, we found that TTK deficiency resulted in increased cell death in both of the tested cell lines (Fig. 3A; arrows in Merged images show non-viable cells having nuclear stain only). Additionally, in both HepG2 KD cell lines, more cells displayed irregular, aggregated and accumulated nuclei, characteristics of dying/dead cells (positive nuclear stain) or senescent cells (positive Calcein-AM and nuclear stains)^11^,^12^, when compared with Ctrl cells (Fig. 3A and Fig. S8A).

We further evaluated senescence, which is an important form of cell death and is a potent tumor suppressive mechanism that occurs *in vivo*^11^,^13^,^14^. Senescent cell death can be characterized by expression of pH-dependent β-galactosidase activity and/or changes in cell shape (typically an enlarged cell size)^12^. As demonstrated in Fig. 3B and Fig. S8B, in contrast to Ctrl cells, TTK KD cells exhibited increased senescence-associated β-galactosidase (SA-β-Gal) activity measured at pH 6, in both HCC cell lines. Moreover, in HepG2 cell line, TTK knockdown also resulted in increased numbers of enlarged viable cells (Fig. 3A).

Next, another important effector mechanism of cell death, namely autophagy^15^,^16^, was examined. Protein levels of LC3-II, a sensitive indicator of autophagy, were analyzed in these cell lines. We found that knockdown of TTK led to significantly heightened levels of LC3-II (Fig. 3C).

The pathways resulting in apoptosis of HepG2 KD cell lines were then analyzed using two different approaches. By Western blotting, we did not detect any levels of cleaved caspaces-3 or -9 in TTK KD cells (data not shown). Using the Annexin V-FITC/PI/Hoechst Apoptosis Assay application by Celigo, overall, only a small fraction of cells (less than 1%) stained positive for Annexin V in both Ctrl and two TTK KD cells (data not shown). Amongst these, TTK KD#1 cells showed significantly increased early apoptosis (Annexin V^+^) (Fig. S9A), when compared to Ctrl cells; whereas there was no difference in overall levels of apoptotic cells (Annexin V^+^PI^+^) (Fig. S9B).

In parallel, Fig. S9C showed that TTK deficiency per se had no substantive impacts on cell proliferation and cell cycle (as indicated by BrdU and DAPI Incorporation by Celigo).

Taken together, these data demonstrate that targeted inhibition of TTK specifically limits liver cancer cell growth promoting greater levels of senescent and autophagic cell death.

### *In vitro* knockdown of TTK blocks intrahepatic growth of HCC xenografts *in vivo*

In order to investigate the *in vivo* anti-cancer activity of TTK inhibition, we established an intrahepatic human xenograft tumor model by infusing HepG2 cells into athymic nude mice via portal vein^17^,^18^ (see more details in Materials and Methods).

**Figure 4:**
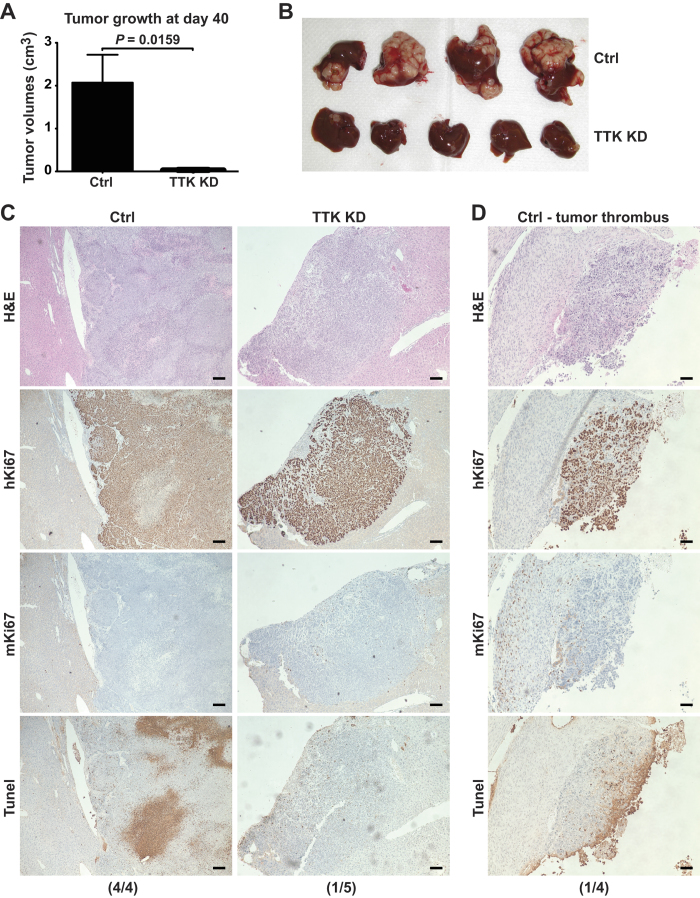
*In vitro* knockdown of TTK blocks *in-vivo* growth of HCC xenografts in the liver. (**A,B**) Ctrl and TTK KD HepG2 cells (0.5 × 10^6^ cells/liver) were infused into nude mice via portal vein. Tumor volumes (**A**) and images of tumor-bearing livers (**B**) are examined on day 40. *n* = 4–5 per group. (**C**) Representative images of pathology confirmation of intrahepatic HCC xenograft tissues by IHC staining of H&E, human Ki67 (hKi67), mouse Ki67 (mKi67), and Tunel. Incidence of pathologically confirmed tumors is shown on the bottom. (**D**) Tumor thrombus noted in one out of four Ctrl mice. Incidence is shown on the bottom. Scale bar, 100 μm Error bars, mean ± s.e.m, Mann-Whitney U test.

We found that, strikingly, intrahepatic growth of TTK KD cells was significantly hindered, as compared with Ctrl cells (Fig. 4A,B). We then performed immunohistochemical staining inclusive of H&E, human Ki67 (hKi67), mouse Ki67 (mKi67), and Tunel using these xenograft tissues (Fig. 4C and Fig. S10 (shown in a higher magnification)). 100% (four out of four) mice that received Ctrl cells had large, actively proliferating tumors (as indicated by positive hKi67 stain), associated with massive tumor necrosis (shown in Tunel staining) due to aggressive tumor growth. In stark contrast, only one out of five (20%) mice implanted with TTK KD cells exhibited a small tumor nodule that could be pathologically noted. This tumor was also very small and localized, and absent of tumor necrosis. More intriguingly, in one of the four Ctrl mice that had aggressive tumors, we found associated minor tumor thrombus (Fig. 4D), which is associated with poor prognosis of liver cancer^19^,^20^.

These results suggest that TTK inhibition may effectively prevent spread of liver cancer cells and may limit intrahepatic recurrence as well as progression of HCC.

### *In vivo* knockdown of TTK limits *in-vivo* growth and aggressiveness of HCC

We explored the therapeutic application of *in-vivo* TTK siRNAs with respect to impacts on HCC growth in the liver using our intrahepatic xenograft model. The Invivofectamine 2.0-siRNA Complex was systematically delivered to mouse liver tissue in a non-invasive manner using low-pressure i.v. injection (more technical and treatment details can be found in Materials and Methods).

**Figure 5:**
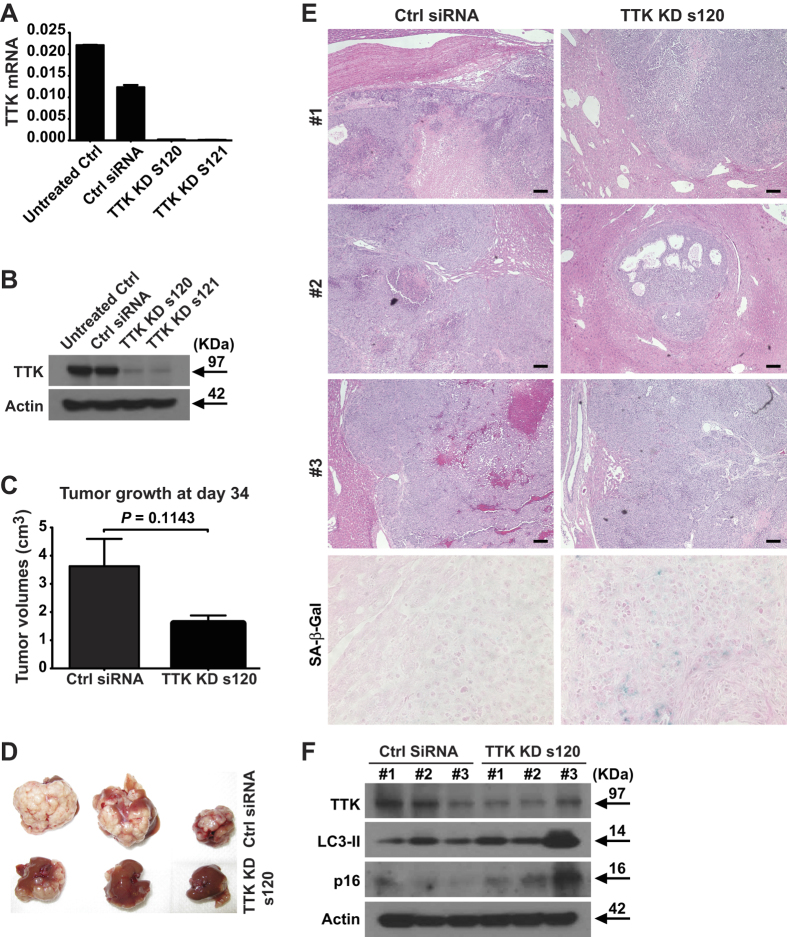
Intrahepatic growth of HCC xenografts is inhibited by *in vivo* knockdown of TTK. (**A,B**) Validation of gene silencing efficiency of the two *in vivo* TTK siRNAs (s120 and s121) by qPCR (**A**) and Western blot (**B**), using HepG2 cells *in vitro*. Negative Ctrl #1 siRNA served as a scramble control. (**C,D**) 0.5 × 10^6^ of HepG2 cells were implanted into nude mice via portal vein infusion. At day 10, TTK siRNA s120 or scramble control siRNA (Ctrl siRNA) were given to tumor-bearing mice (7 mg/kg) using Invivofectamine 2.0 reagent via i.v. injection. Tumor volumes (**C**) and images of tumor-bearing livers (**D**) are examined on day 34. (**E**) H&E pathology confirmation of intrahepatic HCC xenograft tissues and representative images of senescence staining by SA-β-Gal. (**F**) Protein levels of TTK, LC3-II, and p16 in intrahepatic HCC xenograft tissues by immunoblotting. Scale bar, 100 μm. *n* = 3 per group; error bars, mean ± s.e.m, Student’s *t*-test.

Initially, gene silencing efficacy of two Ambion^@^ *In Vivo* Ready siRNAs specifically targeting human TTK were tested using HepG2 cells *in vitro*. Both TTK siRNAs exhibited potent knockdown activity, as shown by qPCR and Western blot (Fig. 5A,B). TTK KD s120 was then chosen for *in vivo* delivery. Tumor volumes were smaller in TTK KD s120 group as compared to the control (Ctrl) siRNA group, showing a trend towards inhibition of *in-vivo* HCC growth by such *in vivo* TTK siRNA (Fig. 5C,D). Subsequent pathology staining provided further clear evidence confirming the *in vivo* anti-tumor activity of *in-vivo* gene silencing of TTK. Specifically, in the Ctrl siRNA group, we found large tumor areas displaying extensive tumor necrosis with inflammatory cell infiltration (Fig. 5E and Fig. S11 (shown in higher magnification)). In addition, one tumor showed massive intratumoral hemorrhage (Fig. 5E and Fig. S11, #3 mouse). In contrast, in the TTK KD s120 group, all tumors were localized and less aggressive, and were accompanied with much less necrotic areas and no hemorrhage (Fig. 5E and Fig. S11).

In parallel, immunohistochemical staining including human Ki67 (hKi67), mouse Ki67 (mKi67), and Tunel were performed to confirm tumor pathology (Figs S12 and S13 (shown in higher magnifications)).

Moreover, as shown in Fig. 5F, decreased TTK protein levels in xenografts from the TTK KD s120 group confirmed the efficacy of such *in-vivo* silencing. This is associated with increased senescence (as indicated by SA-β-Gal staining (Fig. 5E) and p16 (a senescence-related marker) levels (Fig. 5F)) and elevated autophagy (as indicated by LC3-II levels (Fig. 5F)).

Collectively, these data suggest the potential utility of targeted TTK inhibition in limiting growth and spread of HCC.

## Discussion

In our previous work, in a large cohort of liver cancer patients, we demonstrated that tissue-associated TTK could be used for prognosis and potentially risk-stratification of liver cancer^8^. In the current study, we further validate our previous findings using another independent patient cohort, and more importantly, demonstrate a critical role of TTK in liver cancer development and progression.

Tests for TTK in blood and other body fluids of patients with rheumatoid arthritis and osteoarthritis have been done^21^. Elevation of TTK expression has also been reported in other types of cancer, including breast and lung cancer^22^,^23^,^24^. The utility of these measurements increases clinical potential as a non-invasive molecular biomarker for liver cancer. Determination of correlations between markedly elevated circulating TTK levels and intratumoral expression in liver cancer as well as better definition of the role in tumor biology would be the next logical steps in drug development.

The identification of TTK as a novel cancer-testis antigen (CTA)^25^,^26^ allows development as potential target in the setting of anti-cancer immunotherapy, given that expression is restricted mainly to cancer cells^27^,^28^. The tumor specificity of TTK expression is also supported by our data showing total or near absence of TTK in non-neoplastic liver in all liver cancer patients.

There have been also non-specific reaction issues caused by TTK antibodies. Thus, we have used RNAscope ISH assay on limited clinical samples from one liver cancer patient as a trial test. This RNA ISH technology circumvents non-specific reactions^10^. Not only could this test specifically and accurately quantify TTK expression, but allowed visualization of the subcellular localization patterns of TTK gene transcripts in neoplastic cells. Our preliminary RNA ISH results confirmed the predominant tumor-specific expression of TTK observed in a large cohort of liver cancer patients. Presumably, these low-TTK expression albeit still histologically tumor-free tissues show a field effect and suggest early involvement of TTK in hepatocarcinogenesis.

Our work identifies molecular underpinnings by which TTK drives hepatocarcinogenesis, at least in part, through preventing senescent and autophagic cell death and facilitating spread of liver cancer cells. These data are, in part, consistent with a recent study that had a focus on the role of TTK in mediating sorafenib resistance^9^. However, unlike this work by Liang *et al.*^9^, we found that TTK deficiency does not have substantial impacts on liver cancer cell proliferation nor does this intervention impact cell cycle progression.

We further demonstrate in a clinically relevant animal model that intervention strategies that inhibit TTK e.g. gene silencing tools (as used in this study) or chemical compounds^29^,^30^,^31^,^32^,^33^,^34^,^35^ or intracellular antibodies with blocking activity^36^,^37^,^38^,^39^ may effectively curtail cancer cell growth and prevent future spread. Thus TTK inhibition may ultimately limit HCC development, recurrence and progression. Specifically, in our intrahepatic xenograft mouse studies, we show that both *in vitro* and *in vivo* knockdown of TTK efficiently slow tumor growth and decrease aggressiveness of liver cancer cells. In stark contrast, in the TTK-high control group, higher tumor incidence/burden and more aggressive features (e.g. massive necrosis, infiltration of inflammatory cells, and tumor thrombus formation) are noted. These experimental data correlate well with clinical features of liver cancer patients, i.e. patients with low intratumoral TTK levels appear to have a better prognosis, associated with a much less aggressive clinical course. However, the exact molecular mechanisms impacted by TTK that dictate the malignant potential of liver cancer cells remain to be determined.

Two recent clinical trials have been conducted using multiple peptides derived from cancer antigens, inclusive of TTK, in esophageal squamous cell carcinoma (ESCC) and non-small cell lung cancer^40^,^41^. These studies have demonstrated that such cancer vaccination is safe and induces strong specific T cell responses, which are in turn associated with better clinical outcomes^40^,^41^. Therefore, cancer vaccination using TTK peptide provides yet another potential therapeutic approach against liver cancer and other cancers.

Currently, transcatheter arterial chemoembolization (TACE) is the most preferred treatment for the palliation of non-resectable liver cancer. Enhancing the outcome of TACE in these patients by exploring biomarkers in tumor histology is one of the areas of interest in liver cancer treatment. The effect of TTK expression on the outcome of liver-directed treatment option such as TACE and radiofrequency ablation (RFA) in patients with non-resectable liver cancer remains unclear. We propose future studies to investigate the impact of TTK expression on overall survival and response to TACE and RFA in non-resectable liver cancer.

Taken together, in addition to further validating published results, our unique findings provide the pre-clinical pathophysiological “proof of principle” evidence necessary for development of an efficacious, therapeutic approach against liver cancer. Our results provide both clinical evidence and mechanistic data that TTK is a prognostic indicator and may be an effective therapeutic target in HCC appropriate for clinical development. When translated into the clinic, liver cancer patients might be stratified by tumor TTK levels prior to treatment. Therapeutic approaches that inhibit TTK would likely target cancer cells only, circumventing unwanted off-target effects. These proposed treatment modalities are innovative, appear specific, non-toxic and seem potentially more effective to prevent and manage progression, recurrence and death of liver cancer, and, potentially, other types of cancer that express high TTK levels.

## Material and Methods

### Patients and clinical samples

34 pairs of snap-frozen and 18 pairs of formalin-fixed, paraffin-embedded (FFPE) HCC and adjacent noncancerous liver tissue samples were randomly selected retrospectively from patients with hepatitis B virus (HBV)-related HCC who underwent hepatectomy at Peking Union Medical College Hospital (PUMCH) between September 1, 2003 and February 28, 2012. HCC and noncancerous liver tissue samples were determined by pathological review. All pathological images were re-examined by another independent pathologist in a blinded fashion. All patients had pathologically confirmed HCCs (which was done immediately after surgery) and did not receive any anti-cancer treatment prior to surgery. Fresh tissue samples were collected in the operating room and processed immediately within 15 minutes after resection. Snap-frozen tissues were stored at −80 °C for subsequent Western blot analysis of expression levels of TTK protein. Detailed clinicopathological information is listed in Supplementary Table 1. In addition, RNA ISH technology requires freshly made FFPE tissue blocks. Thus, another pair of FFPE HCC and noncancerous liver tissue samples was obtained from a HBV-HCC patient who underwent hepatectomy at PUMCH on February 3, 2015. The pathological classification of this tumor tissue is moderately differentiated and the largest diameter is 5.5 cm. All human studies were done at PUMCH in China. The study was approved by the Ethics in Research Committee of PUMCH. Written informed consent was obtained from all study participants before surgery and the methods were performed in according with the approved guidelines.

### Tumor cell lines

Human HCC cell lines including HepG2, Hep3B, PLC/PRF/5 and SK-HEP-1 cells were obtained from American Type Culture Collection (ATCC, Manassas, VA) and maintained in a 37 °C incubator with 5% CO_2_ humidified air in Eagle’s Minimum Essential Medium (EMEM) supplemented with 10% (v/v) fetal bovine serum (FBS, Life Technologies, Carlsbad, CA). Huh7 cells were maintained in Dulbecco’s minimal modified Eagle’s medium (DMEM) as described previously^42^.

### Antibodies and reagents

Mouse anti-β-actin monoclonal antibody (Cat#ab6276) was purchased from Abcam (Cambridge, MA). Rabbit antibody against LC3B (Cat#2775) was from Cell Signaling Technology (Danvers, MA). Rabbit anti-human TTK (C-19) antibody (Cat#sc-540) and mouse antibody against p16 (Cat#sc-1661) were obtained from Santa Cruz Biotechnology, Inc. (Dallas, TX). All chemicals were obtained from Sigma-Aldrich (St Louis, MO), unless otherwise stated.

### Generation of TTK deficient HCC cell lines

Generation of TTK deficient HCC cell lines was performed as previously described with slight modifications^43^,^44^. Briefly, HepG2, Huh7, Hep3B, PLC/PRF/5, or SK-HEP-1 cells were infected separately with an empty shRNA vector control (pLKO.1-puro), or four different human TTK shRNAs (#1: NM-003318.3-522s1c1, TRCN0000006358; #2: NM-003318.3-694s1c1, TRCN0000006356; #3: NM-003318.3-1479s1c1, TRCN0000006357; and #4: NM-003318.3-2515s1c1, TRCN0000011012; Sigma-Aldrich) lentiviral transduction particles (Life Technologies), according to the manufacturer’s instructions. 2 × 10^6^ of 293FT cells were seeded in a 60-mm culture dish in DMEM without antibiotics. The next day, cells reaching 90–95% confluence were cotransfected with 1 μg of shRNA plasmids and 3 μg of ViraPower^TM^ Packaging Mix (an optimized proprietary mix of three plasmids, pLP1, pLP2, and pLP/VSVG from Life Technologies) using 12 μl of Lipofectamine 2000 (Life Technologies) in 1 ml of Opti-MEM I Reduced Serum Medium (Life Technologies). 6 hours later, medium containing the DNA-Lipofectamine^TM^ 2000 complexes was replaced with regular growth medium without antibiotics, and cells were incubated for additional 72 hours. Conditioned cell culture media containing recombinant lentiviral particles were harvested, centrifuged to remove pellet debris, filtered through a 0.45 μm syringe filter, aliquoted, and used immediately or stored at −80 °C.

To generate stable cell lines encoding empty vector shRNA or TTK shRNA, HCC cells were seeded in a 12-well plate and grown for 24 hours in order to reach a confluence of 30–40%. Cells were then infected with above cell culture supernatant containing lentiviral particles (500 μl of lentiviral particles and 500 μl of antibiotics-free regular growth medium) in the presence of 8 mg/ml of Polybrene^®^ (hexadimethrine bromide; Cat#H9268, Sigma-Aldrich). 24 hours later, cells were replenished with regular growth media and cultured for additional 48 hours to allow the expression of selection marker, and then selected with puromycin (a kill-curve assay was performed for each cell line to decide the optimal puromycin concentration: 2 μg/ml for HepG2, Huh7 and Hep3B cells, and 1 μg/ml for PLC/PRF/5 and SK-HEP-1 cells) for at least 10 days. qPCR and Western blot analyses were performed to confirm diminished TTK expression in selected cell lines. Hep3B and PLC/PRF/5 KD#1 and #2 cells did not survive under the selection pressure.

### Assessment of cell viability

Cells (5 × 10^3^) were seeded into 96-well plates. 24, 48, 72, or 96 hours later, cell viability was analyzed using Cell Counting Kit-8 (CCK-8, Cat#CK04-13, Dojindo Molecular Technologies Inc., Rockville, MD) that measures the activity of cellular dehydrogenases, following the manufacturer’s instructions as previously described^17^,^43^,^45^.

### *In situ* cellular analysis

5 × 10^3^ cells were seeded into BD Falcon 353219 Black 96-well plates and grown for 24, 48, 72, or 96 hours. Cell growth was evaluated using the Celigo Cytometer (Nexcelom Bioscience LLC., Lawrence, MA). Bright field images of live cells were captured using the Celigo Cell Counting application as described previously^17^,^43^.

In parallel, cells were also co-stained using the Calcein AM/Hoechst/PI Viability Kit (Cat# CS1-0114, Nexcelom Bioscience LLC.), following the manufacturer’s instructions. Specifically, Calcein AM detects viable, metabolically-active (green fluorescent) cells; propidium iodide (PI) stains non-viable, dead (red fluorescent) cells with compromised membranes; and Hoechst 33342 counterstains nucleated (blue fluorescent) cells. Fluorescent images of stained cells were captured by Celigo Cytometer.

### Apoptosis and cell cycle assays

1 × 10^4^ cells were seeded into BD Falcon 353219 Black 96-well plates and grown for 24 hours.

Cells were washed with PBS twice and incubated with Annexin V-FITC, PI and Hoechst33258 for 30 minutes. Apoptosis was evaluated using the Annexin V-FITC/PI/Hoechst Apoptosis Assay application by Celigo Cytometer (Nexcelom Bioscience LLC.).

Cells were cultured with 10 μM BrdU for 2 hours and cell cycle was analyzed using the BrdU and DAPI Incorporation application by Celigo Cytometer (Nexcelom Bioscience LLC.).

### 3D soft agar colony formation assay

Control and TTK KD cells were trypsinized and washed with PBS. 5 × 10^3^ cells were suspended in DMEM containing agarose (0.4%) and puromycin (2 μg/ml) and layered onto a 1.5-ml solidified bed of DMEM containing 0.6% agar in 6-well plates. Fresh growth media containing puromycin were replaced (1.0 ml/well) twice every week. Three weeks later, colonies were stained with Crystal Violet Reagent (0.005% in PBS; 0.5 ml/well) at room temperature for 1 hour. Crystal Violet Reagent was then removed by washing three times with PBS and blue colonies were counted and photographed using a Nikon microscope or a digital Nikon camera.

### Migration and invasion assays

For migration assay, 5 × 10^4^ cells were plated in the upper chamber (Corning, 3422) with serum-free EMEM culture media. 600 μl of EMEM containing 20% FBS was added into the lower chamber. 24 hours later, cells migrated to the opposite side of the upper chamber were fixed and stained with 0.1% crystal violet.

For invasion assay, 1.5 × 10^5^ cells were seeded in the matrigel-coated upper chamber with serum-free EMEM and the concentration of matrigel matrix (BD Biosciences, San Jose, CA) is 1 mg/ml. 600 μl of EMEM containing 20% FBS was added to the lower chamber. 72 hours later, the invaded cells were fixed and stained with 0.1% crystal violet.

The images were photographed using a Nikon microscope.

### β-galactosidase staining assay

β-galactosidase staining was performed using Senescence β-Galactosidase Staining Kit (Cat#9860, Cell Signaling Technology)^13^, following the manufacturers’ instructions. Briefly, HCC cells grown in 6-well plates or frozen sections from xenograft tissues were fixed with Fixative Solution for 15 minutes at room temperature, and then incubated with β-Galactosidase Staining Solution at 37 °C in a dry incubator without CO_2_ overnight (cell lines) or 48 hours (frozen sections). Slides were counterstained with Eosin. Cells or slides were examined under a Nikon microscope for the development of blue color.

### Western blot analysis

Snap-frozen tissue samples or cultured cells were lysed in ice-cold modified RIPA buffer containing 50 mM Tris-HCl (pH 7.4), 150 mM NaCl, 0.5% sodium deoxycholate, 0.1% SDS, 1% NP-40, and Pierce^TM^ Protease and Phosphatase Inhibitor Mini Tablets (Cat#88668, Thermo Scientific, Rockford, IL). The lysates were sonicated briefly on ice and centrifuged at 14,000 rpm for 10 minutes at 4 °C. The measurement of protein concentrations and detailed immunoblotting procedures were carried out as described previously^17^,^43^,^46^.

### Real-time quantitative PCR (qPCR)

Total RNA were extracted and purified from tissues or cells using an RNeasy Mini kit (Cat#74104, QIAGEN Sciences, Germantown, MD). Reverse transcription was conducted on 1 μg of total RNA using BIO-RAD iScript^TM^ cDNA Synthesis Kit (Cat#170-8891, Bio-Rad Laboratories, Hercules, CA). Real-time quantitative PCR (qPCR) analysis was performed as previously described^8^. β-actin served as an internal control and relative expression was calculated using 2^−ΔΔCT^ values. The specificity of primers for human TTK and β-actin has been validated; sequences and PCR conditions were used exactly as published previously^8^. The primer sequences were: β-actin (forward), CTCTTCCAGCCTTCCTTCCT; β-actin (reverse), GCACTGTGTTGGCGTACAG; TTK (forward), AGCAGCAACAGCATCAAATACT; TTK reverse, GCTTGAACCTCCACTTCCTATC. All primers were obtained from Life Technologies.

### *In vivo* silencing of TTK in intrahepatic xenograft models

In this study, we established an intrahepatic xenograft model potentially more relevant for human liver cancer when compared to more commonly used subcutaneous xenograft models. The reasons are the following: recurrent liver cancer comprises patient groups with intrahepatic spread; liver cancer cells have the propensity to grow in the hepatic milieu. HCC metastasis is rarely found in connective tissues and subcutaneous locations. The intrahepatic human xenograft tumor model was established as previously described^18^ with modifications. Briefly, on day 0, subcultures of HepG2 cells were harvested by trypsinization and resuspended with Hanks’ balanced salt solution containing 2% fetal bovine serum to obtain single-cell suspension for injection. Male nude mice (NU/J mice, Stock#002019; Jackson Laboratory, Bar Harbor, ME) of 8 weeks old were injected with 0.5 × 10^6^ HepG2 cells/0.2 ml directly into liver through the portal vein. On day 10, tumor-bearing mice received either TTK Silencer Select Validated siRNA (ID: s120, Cat#4407267) or Silencer Select Negative Control #1 siRNA (Cat#4404020) (at a dose of 7 mg/kg) via intravenous (i.v.) injection using low pressure. This will systematically deliver *In Vivo* siRNA to mouse liver tissue in a non-invasive manner. Invivofectamine 2.0 (Life Technologies), a proprietary, animal-origin-free lipid based *in vivo* RNAi transfection reagent designed for systemic siRNA delivery with high *in vivo* transfection efficiency in liver following i.v. injection, was used. The Invivofectamine 2.0-siRNA Complex was prepared according to the manufacturer’s instructions (Life Technologies). On day 34, mice were euthanized for tissue harvesting and tumor examination, followed by pathological characterization of HCC xenografts. The experimental procedures were approved by institutional animal care and use Committee at Beth Israel Deaconess Medical Center. The methods were carried out in according with the approved guidelines.

### Immunohistochemistry

Immunohistochemistry procedures were executed as previously described^45^,^46^,^47^, using FFPE tissue blocks from HBV-HCC patients or FFPE and frozen sections from xenograft tissues^18^,^45^,^47^.

### RNA *in situ* hybridization (ISH) assay

The TTK ISH was performed using the RNAscope (Brown) FFPE kit (Advanced Cell Diagnostics, Hayward, CA) according to the manufacturer’s instructions. Briefly, FFPE tissues were cut in 4 μm thick sections. The sectioned slide was baked in a dry oven for 1 hour at 60 °C, de-paraffinized by fresh xylene and 100% ethanol, and then dried at room temperature. Dried tissue sections were treated with Pretreat 1 solution and were boiled at 100 °C for 15 minutes in Pretreat 2 solution. When boil time is over, the hot slides were immediately put into distilled water and then dried at room temperature. The next day, to eliminate the RNA binding proteins, tissue sections were treated with Pretreat 3 solution (protease treatment) and hybridized with the provided probes for human PPIB (as a positive control), DapB (as a negative control) and TTK at 40 °C for 2 hours. TTK probe was designed based on 1229–2294 of human TTK (NM_003318.4) targeting the overlapping region of two transcript variants. After washes with provided washing buffer, the probes were detected by AMP1-6 solution and colorized using diaminobenzidine solutions A and B, followed by counterstaining using 50% hematoxylin and 0.02% ammonia.

### Statistical analysis

All data are represented as median with range or mean ± standard error (s.e.m). For statistical analyses, Student’s t-test or Mann-Whitney U test or One-way ANOVA was performed when appropriate. Significance was defined as *P* < 0.05. All statistical analyses were performed using SPSS 17.0 software (SPSS Inc., Chicago, IL).

## Additional Information

**How to cite this article**: Miao, R. *et al.* Utility of the dual-specificity protein kinase TTK as a therapeutic target for intrahepatic spread of liver cancer. *Sci. Rep.* **6**, 33121; doi: 10.1038/srep33121 (2016).

## Supplementary Material

Supplementary Information
