# Supplementary material for: Utility of the dual-specificity protein kinase TTK as a therapeutic target for intrahepatic spread of liver cancer
Source: Sci Rep. 2016 Sep 13;6:33121. doi: 10.1038/srep33121 (PMC5020615; doi:10.1038/srep33121)

Utility of the dual-specificity protein kinase TTK as a therapeutic target for intrahepatic spread of liver cancer

Ruoyu Miao,[1,2*] Yan Wu,[2*] Haohai Zhang,[1] Huandi Zhou,[3] Xiaofeng Sun,[2] Eva Csizmadia,[2] Lian He,[1] Yi Zhao,[4] Chengyu Jiang,[3] Rebecca A. Miksad,[5] Tahereh Ghaziani,[2] Simon C. Robson[2†] and Haitao Zhao[1†].

[1]Department of Liver Surgery, Peking Union Medical College Hospital, Chinese Academy of Medical Sciences and Peking Union Medical College, Beijing 100730, China.

[2]Liver Center and The Transplant Institute, Department of Medicine, Beth Israel Deaconess Medical Center, Harvard Medical School, Boston, MA 02115, USA.

[3]State Key Laboratory of Medical Molecular Biology, Institute of Basic Medical Sciences, Chinese Academy of Medical Sciences and Peking Union Medical College, Beijing 100005, China

[4]Key Lab of Intelligent Information Processing of Chinese Academy of Sciences, Institute of Computing Technology, Beijing 100190, China

[5]Division of Hematology/Oncology, Department of Medicine, Beth Israel Deaconess Medical Center, Harvard Medical School, Boston, MA 02115, USA.

[*] These authors contributed equally to the work.

[†]Corresponding authors. Email: ZhaoHT@pumch.cn (H.Z.); srobson@bidmc.harvard.edu (S.C.R.)

SUPPLEMENTARY TABLES

Table S1. Clinical characteristics of HBV-HCC patients.

Clinical Variable		Western Blot (n = 34)		IHC (n = 18)	
		No.	%	No.	%
Age	≤55 years	15	44.1%	8	44.4%
	>55 years	19	55.9%	10	55.6%
Gender	Female	5	14.7%	1	5.6%
	Male	29	85.3%	17	94.4%
HBV	HBsAg-	8	23.5%	2	11.1%
	HBsAg+HBeAg-	18	52.9%	13	72.2%
	HBsAg+HBeAg+	8	23.5%	3	16.7%
HBsAg	Negative	8	23.5%	2	11.1%
	Positive	26	76.5%	16	88.9%
ALT	≤40 U/L	25	73.5%	5	27.8%
	>40 U/L	9	26.5%	13	72.2%
Bilirubin	≤17 μmol/L	16	47.1%	10	55.6%
	>17 μmol/L	18	52.9%	8	44.4%
Albumin	<35 g/L	5	14.7%	3	16.7%
	≥35 g/L	29	85.3%	15	83.3%
AFP	≤20 ng/mL	11	32.4%	7	38.9%
	>20 ng/mL	23	67.6%	11	61.1%
Cirrhosis	No	6	17.6%	1	5.6%
	Yes	28	82.4%	17	94.4%
Edmondson grade	I	10	29.4%	6	33.3%
	II	16	47.1%	6	33.3%
	III/IV	8	23.5%	6	33.3%
Tumor number	Solitary	25	73.5%	10	55.6%
	Multiple	9	26.5%	8	44.4%
Tumor size	≤5 cm	18	52.9%	11	61.1%
	>5 cm	16	47.1%	7	38.9%
Tumor encapsulation	Complete	26	76.5%	13	72.2%
	Absent/incomplete	8	23.5%	5	27.8%
Satellite	No	27	79.4%	15	83.3%
	Yes	7	20.6%	3	16.7%
Vascular invasion	No	27	79.4%	14	77.8%
	Yes	7	20.6%	4	22.2%

HBV, hepatitis B virus; HBsAg, hepatitis B surface antigen; HBeAg, hepatitis B e antigen; ALT, alanine aminotransferase; AFP, alpha-fetoprotein.

SUPPLEMENTARY FIGURE LEGENDS

fig. S1. Evaluation of TTK protein levels in human HCC tissues. Comprehensive Western blot images of TTK in 34 pairs of matched snap-frozen cancer and non-malignant tissue specimens of HCC patients (n = 34).

fig. S2. Pathological images of biopsies from study subjects with HCC. The different histological grades indicative of tumor differentiation are shown on the right axis. Scale bar, 20 μm. n = 18.

fig. S3. Expression of TTK gene transcripts in human HCC tissues. RNA ISH assessment of TTK gene expression. Two representative images are shown for each sample. Scale bar, 20 μm.

fig. S4. TTK expression at both mRNA and protein levels in various human HCC cell lines. (**A**) mRNA expression of TTK. (**B**) Western blotting and densitometric analysis of TTK protein expression. Error bars, mean ± s.e.m. AU, arbitrary units.

fig. S5. Validation of TTK knockdown in HepG2 and Huh7 cells by qPCR. n = 3; error bars, mean ± s.e.m, Student's t-test. **$P < 0.01$; ***$P < 0.0001$.

fig. S6. Decreased growth of HCC cells caused by TTK deficiency. Representative bright field images of live cells by Celigo Cell Counting application. n = 8.

fig. S7. Migration and invasion assays of HepG2 TTK KD cells. (**A-B**) Quantifications of migratory (**A**) and invasive capacity (**B**) of TTK KD cells. Data are presented as relative to Ctrl cells. n = 3-8; error bars, mean ± s.e.m, One-way ANOVA. $**P < 0.01$; $***P < 0.0001$.

fig. S8. Increased senescence in TTK KD cells. (**A-B**) Quantifications of cellular senescence by Celigo in situ cellular analysis (**A**) and SA-β-Gal activity measurement (**B**). Data are presented as relative to Ctrl cells (**A**) or absolute number of SA-β-Gal^+ cells per counting field (**B**). n = 3; error bars, mean ± s.e.m, One-way ANOVA. $*P < 0.05$; $**P < 0.01$; $***P < 0.0001$.

fig. S9. Apoptosis and cell cycle analyses of HepG2 TTK KD cells. (**A-B**) Population of early apoptotic (Annexin V^+) (**A**) and total apoptotic (Annexin V^+PI^+) (**B**) TTK KD cells by Celigo Apoptosis application. Data are presented as relative to Ctrl cells. (**C**) Proliferation and cell cycle analysis of TTK KD and Ctrl cells by Celigo Cell Cycle application. n = 3; error bars, mean ± s.e.m, One-way ANOVA. $**P < 0.01$.

fig. S10. Representative immunopathological images of intrahepatic HCC xenografts (Ctrl and TTK KD HepG2 cells), human Ki67 (hKi67), mouse Ki67 (mKi67), and Tunel. Scale bar, 40 μm. n = 4-5 per group.

fig. S11. Intrahepatic HepG2 HCC xenograft tissues from tumor-bearing mice. Experimental animals received Ctrl siRNA or TTK siRNA s120. Scale bar, 40 μm. *n* = 3 per group.

fig. S12. Representative immunohistological images of intrahepatic HCC xenograft tissues from tumor-bearing mice received Ctrl siRNA or TTK siRNA s120 by IHC of human Ki67 (hKi67), mouse Ki67 (mKi67), and Tunel. Scale bar, 100 μm. *n* = 3 per group.

fig. S13. Representative images of pathology staining of intrahepatic HCC xenografts from tumor-bearing mice received Ctrl siRNA or TTK siRNA s120 by IHC of human Ki67 (hKi67), mouse Ki67 (mKi67), and Tunel. Scale bar, 40 μm. *n* = 3 per group.

fig. S1

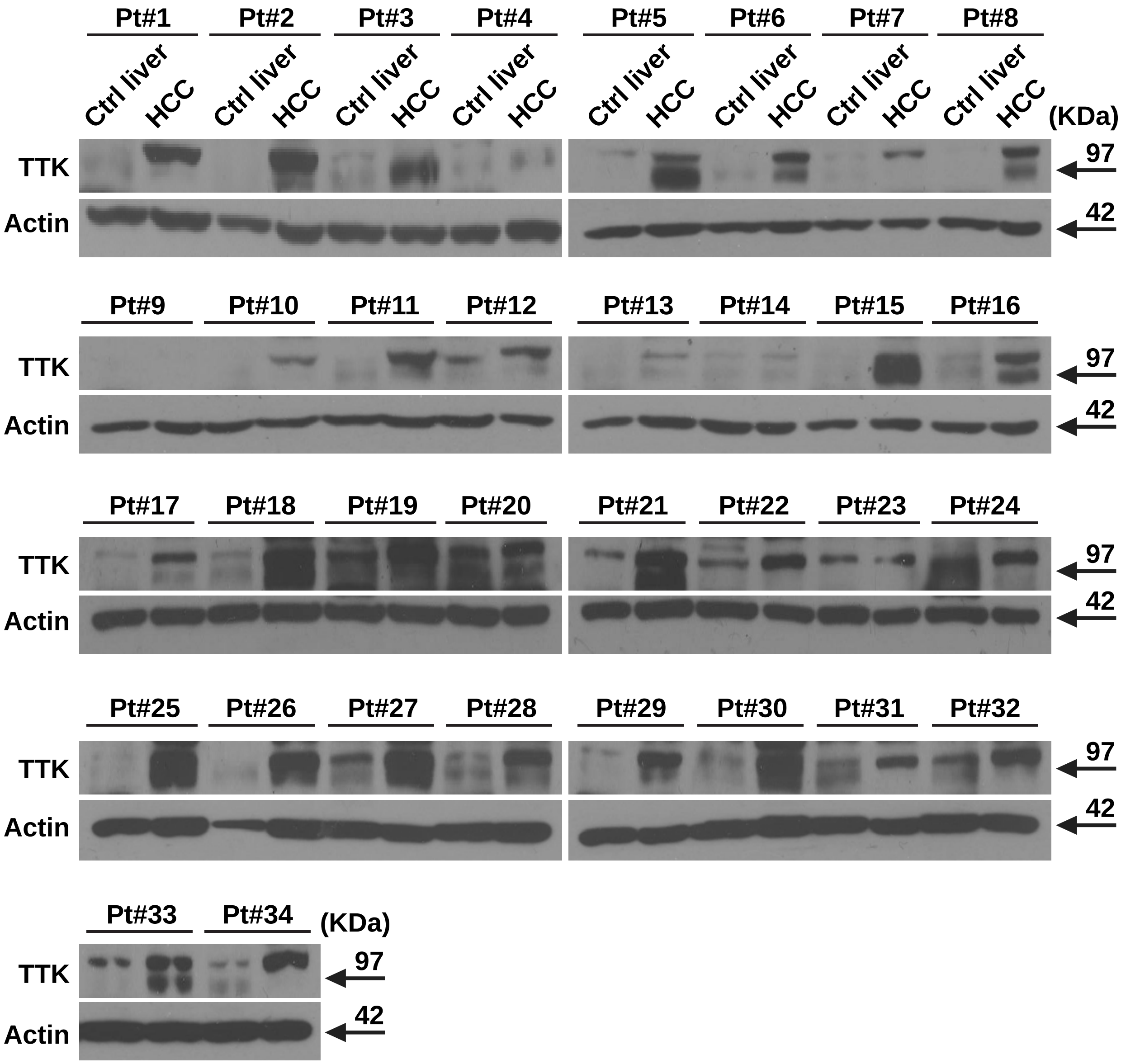
Pt#1
Pt#2
Pt#3
Pt#4
Pt#5
Pt#6
Pt#7
Pt#8
Ctrl liver
HCC
(KDa)
TTK
Actin
97
42
Pt#9
Pt#10
Pt#11
Pt#12
Pt#13
Pt#14
Pt#15
Pt#16
Pt#17
Pt#18
Pt#19
Pt#20
Pt#21
Pt#22
Pt#23
Pt#24
Pt#25
Pt#26
Pt#27
Pt#28
Pt#29
Pt#30
Pt#31
Pt#32
Pt#33
Pt#34

fig. S2

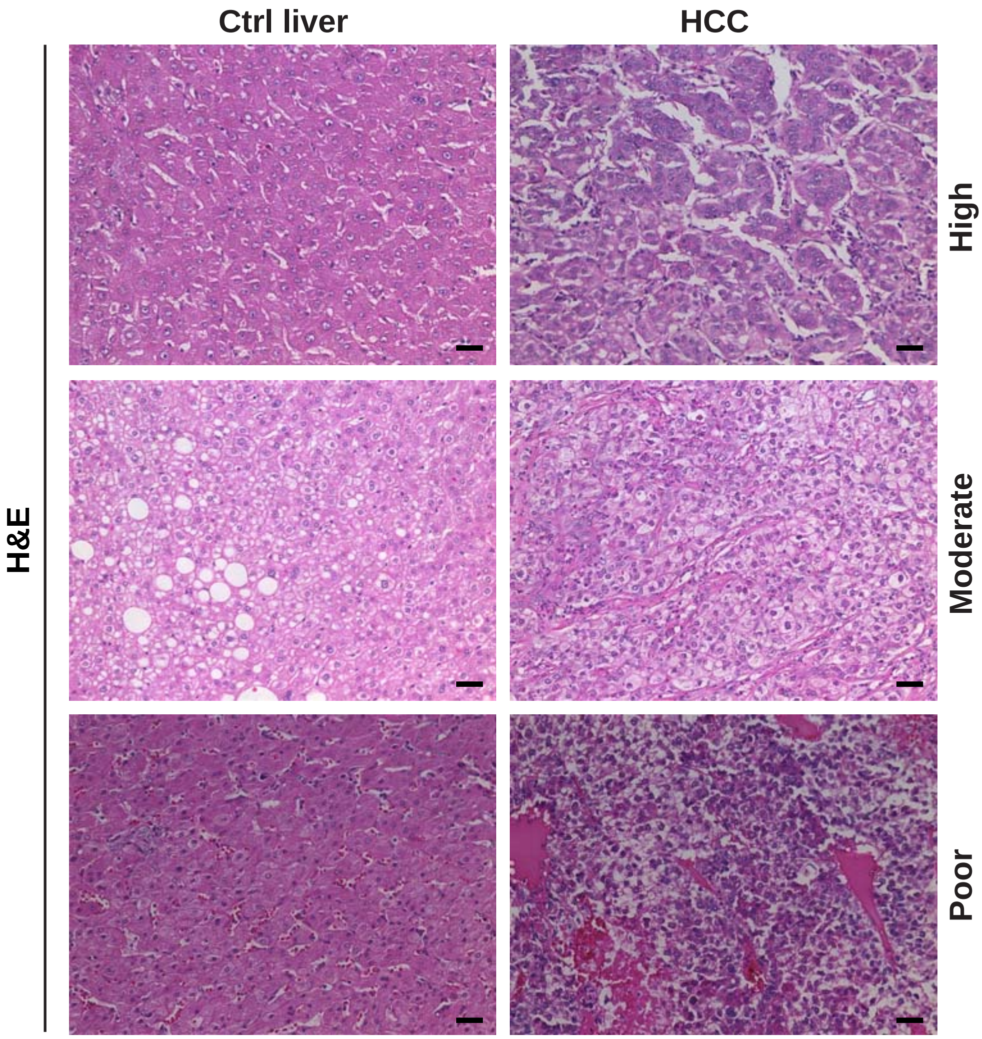

fig. S3

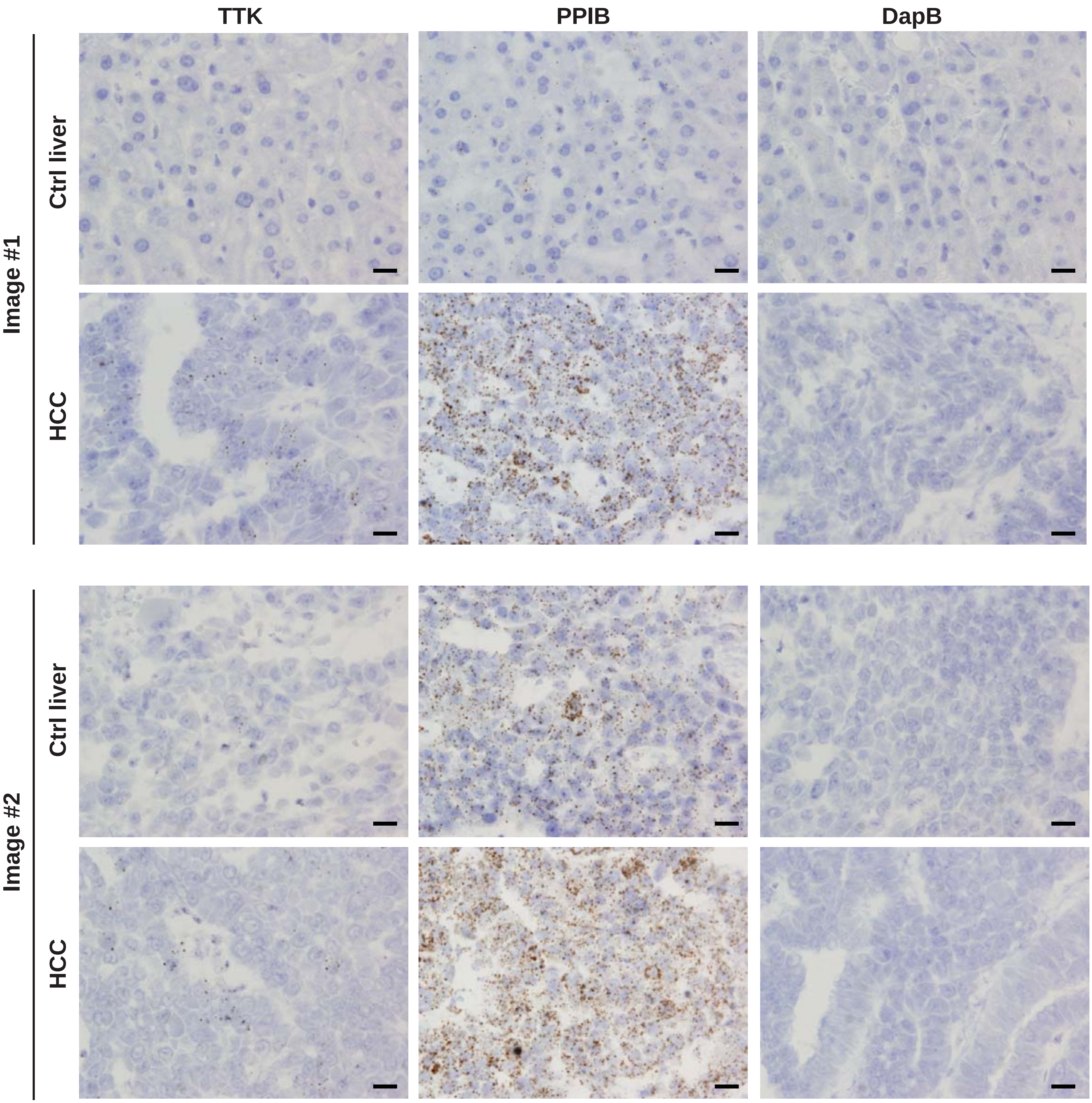

fig. S4

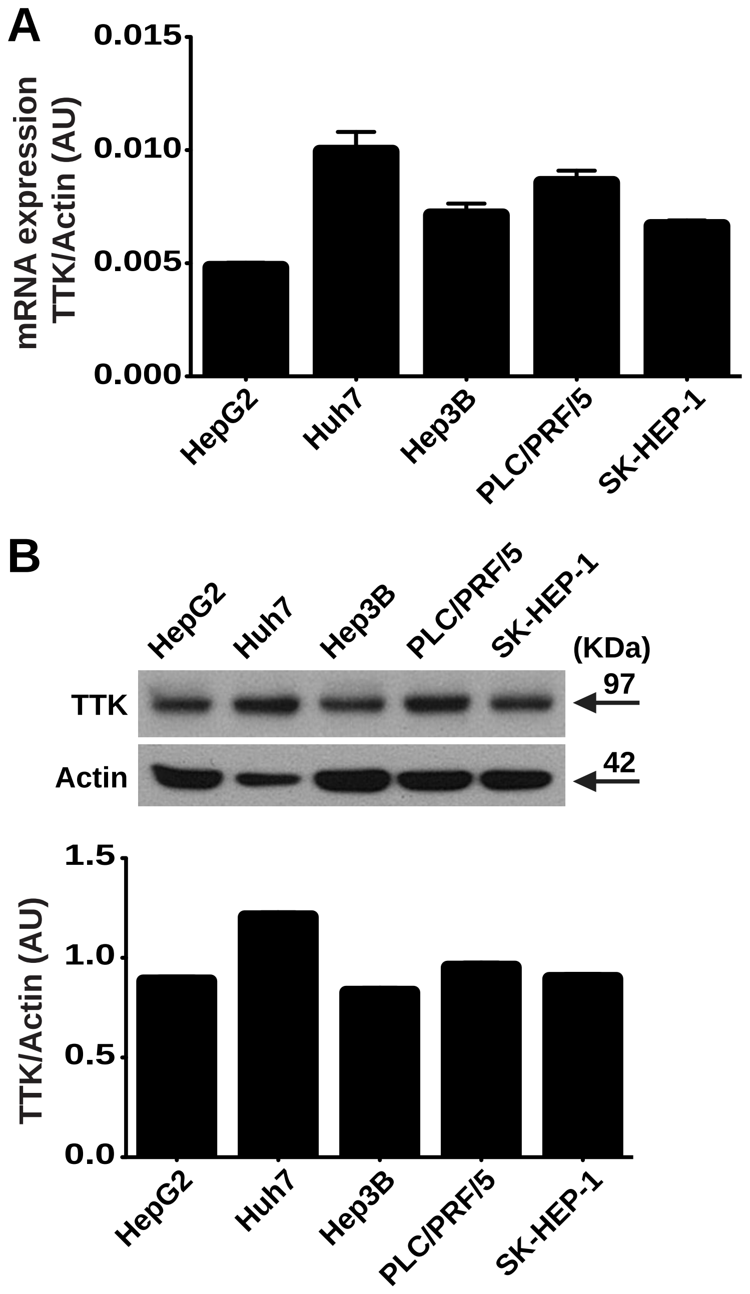

A
mRNA expression
TTK/Actin (AU)
0.015
0.010
0.005
0.000
HepG2
Huh7
Hep3B
PLC/PRF/5
SK-HEP-1
B
HepG2
Huh7
Hep3B
PLC/PRF/5
SK-HEP-1
(KDa)
TTK
97
Actin
42
TTK/Actin (AU)
1.5
1.0
0.5
0.0
HepG2
Huh7
Hep3B
PLC/PRF/5
SK-HEP-1

fig. S5

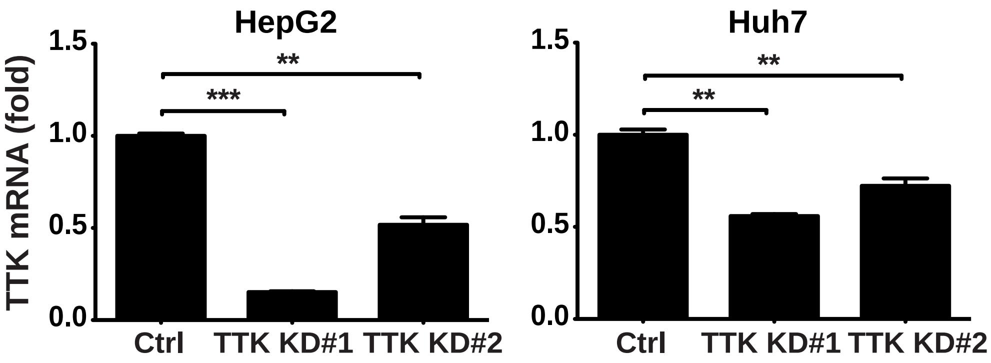

fig. S6

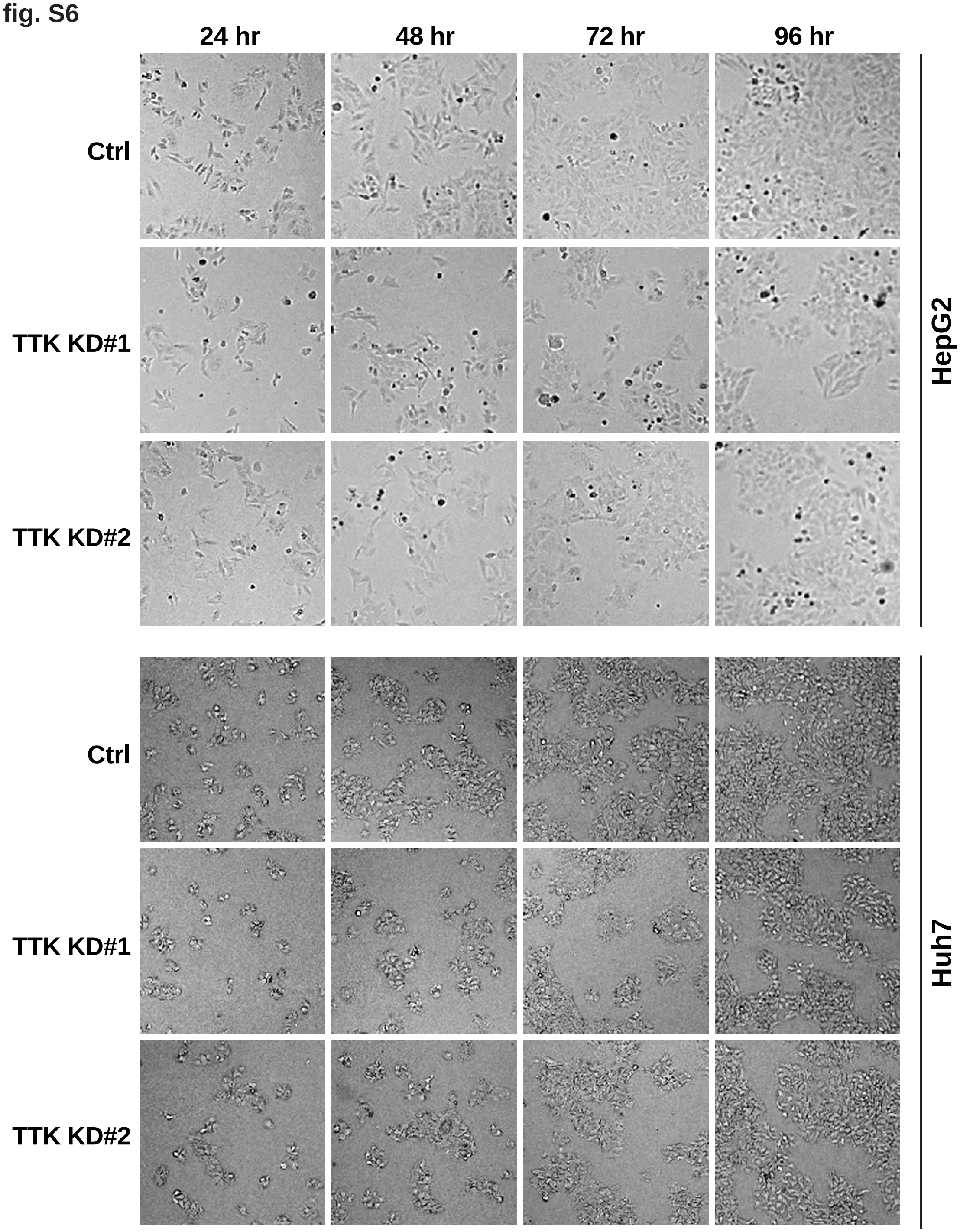

24 hr
48 hr
72 hr
96 hr
Ctrl
TTK KD#1
TTK KD#2
HepG2
Ctrl
TTK KD#1
TTK KD#2
Huh7

fig. S7

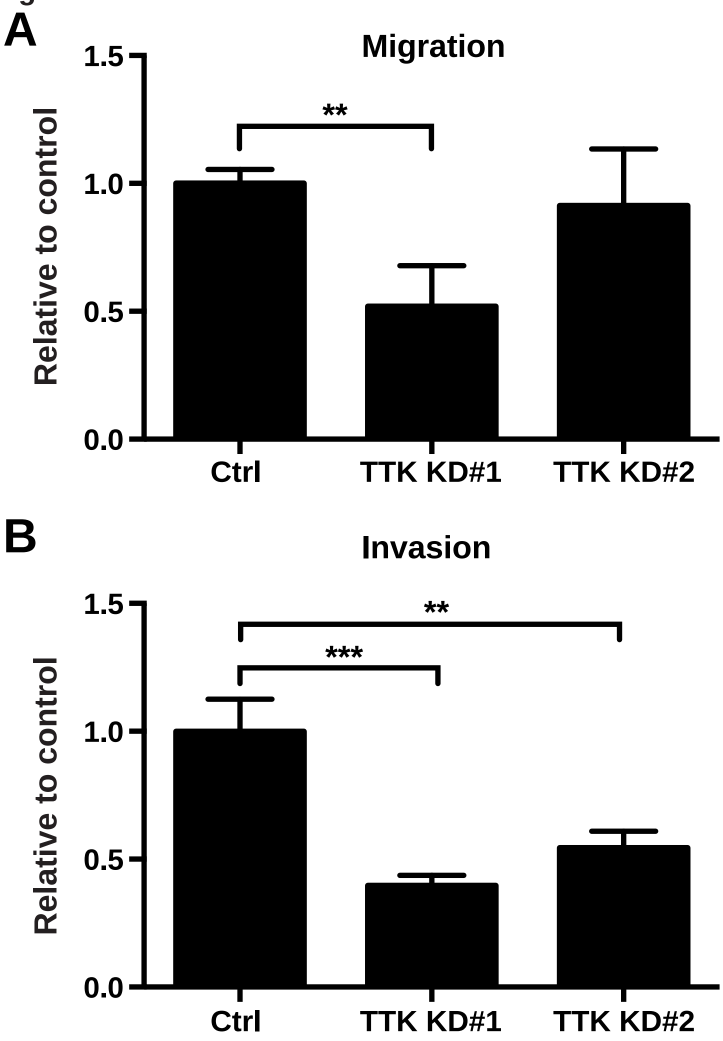
A
Migration
1.5
1.0
0.5
0.0
Relative to control
**
Ctrl
TTK KD#1
TTK KD#2
B
Invasion
1.5
1.0
0.5
0.0
Relative to control
**
***
Ctrl
TTK KD#1
TTK KD#2

fig. S8

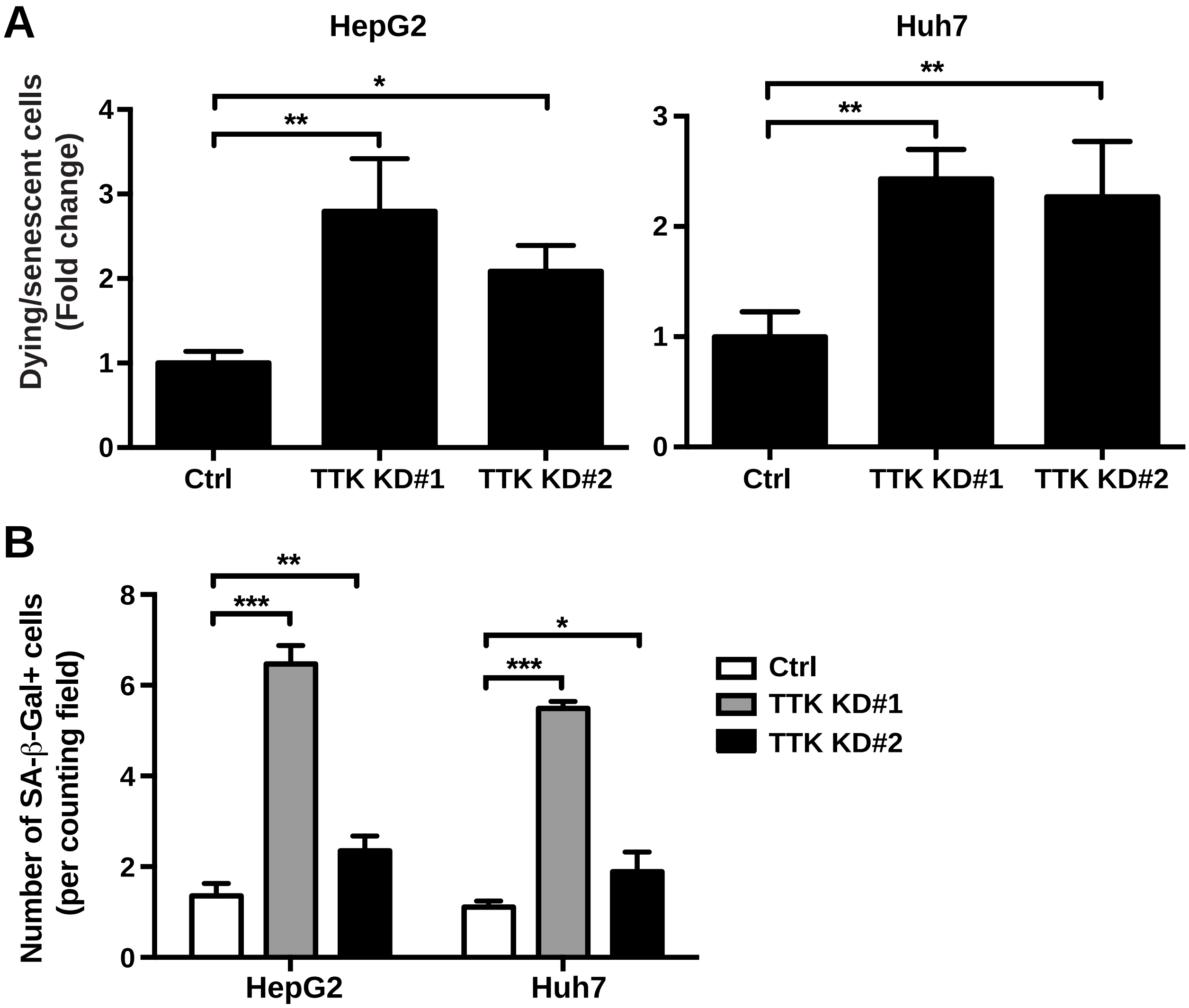
A
HepG2
Huh7
Dying/senescent cells (Fold change)
0
1
2
3
4
*
**
Ctrl
TTK KD#1
TTK KD#2
B
Number of SA-β-Gal+ cells (per counting field)
0
2
4
6
8
**
***
*
***
HepG2
Huh7
Ctrl
TTK KD#1
TTK KD#2

fig. S9

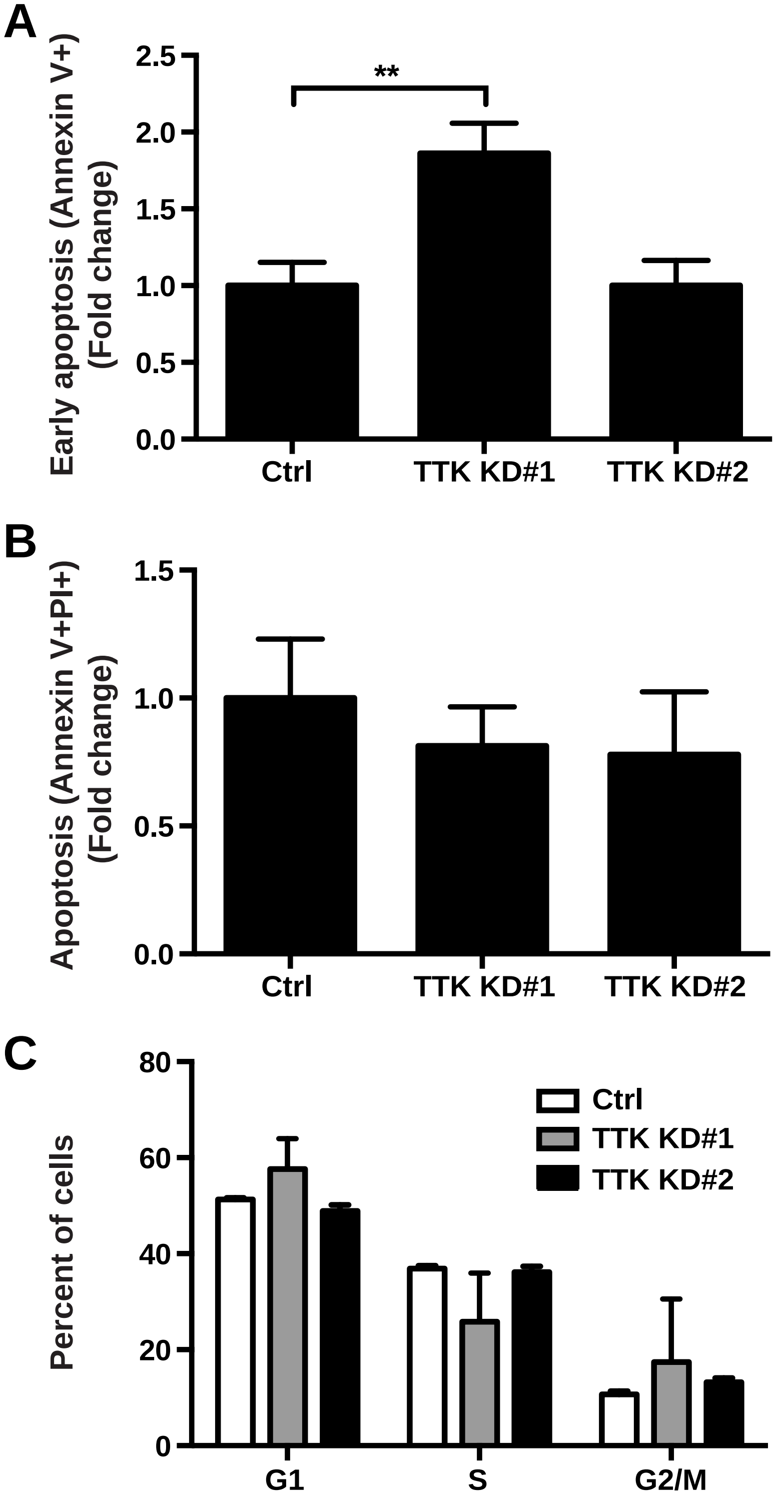
A
2.5
2.0
1.5
1.0
0.5
0.0
**
Early apoptosis (Annexin V+) (Fold change)
Ctrl
TTK KD#1
TTK KD#2
B
1.5
1.0
0.5
0.0
Apoptosis (Annexin V+PI+) (Fold change)
Ctrl
TTK KD#1
TTK KD#2
C
80
60
40
20
0
Percent of cells
Ctrl
TTK KD#1
TTK KD#2
G1
S
G2/M

fig. S10

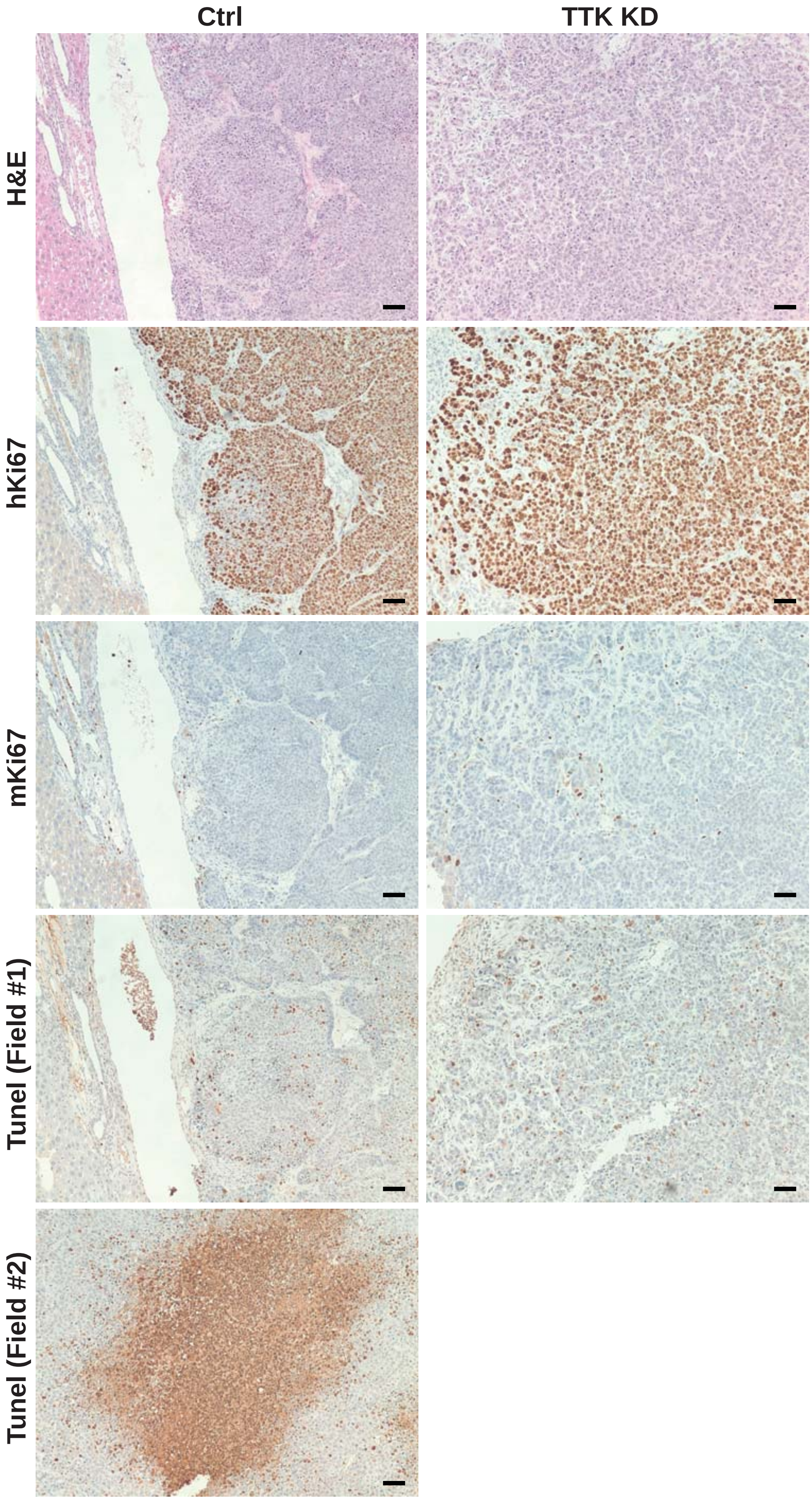

fig. S11

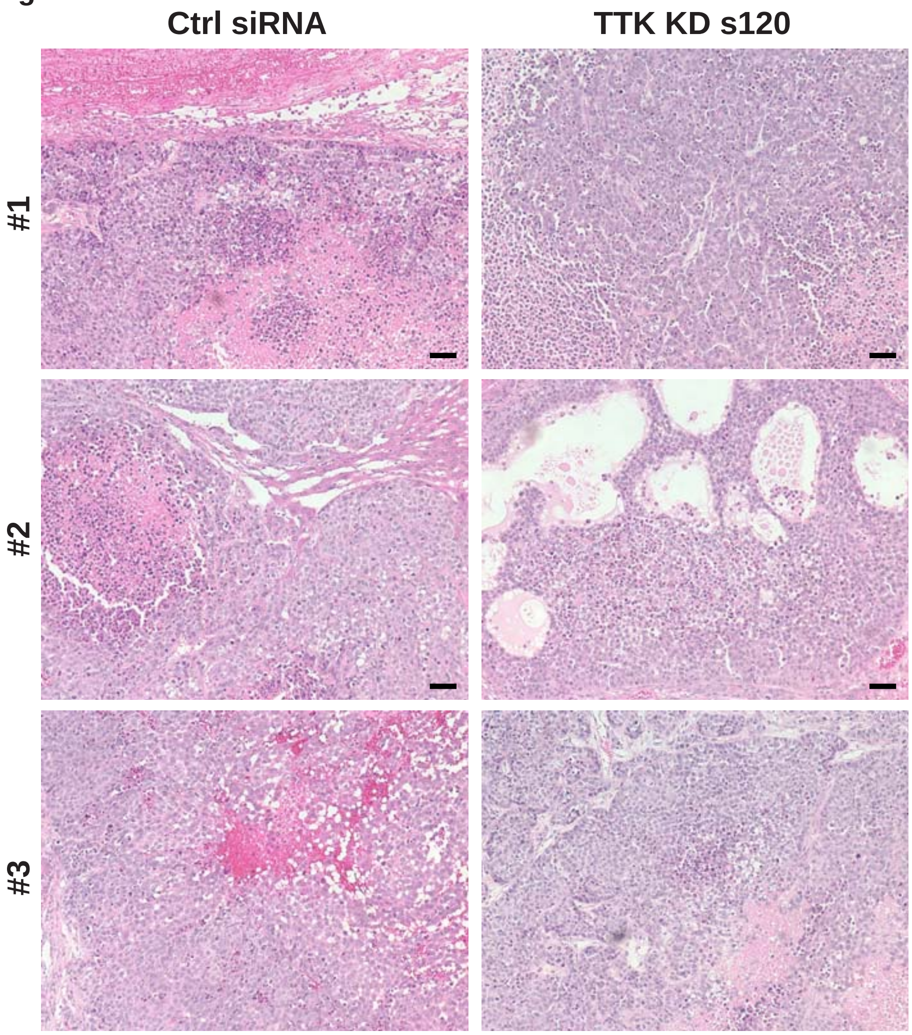

fig. S12

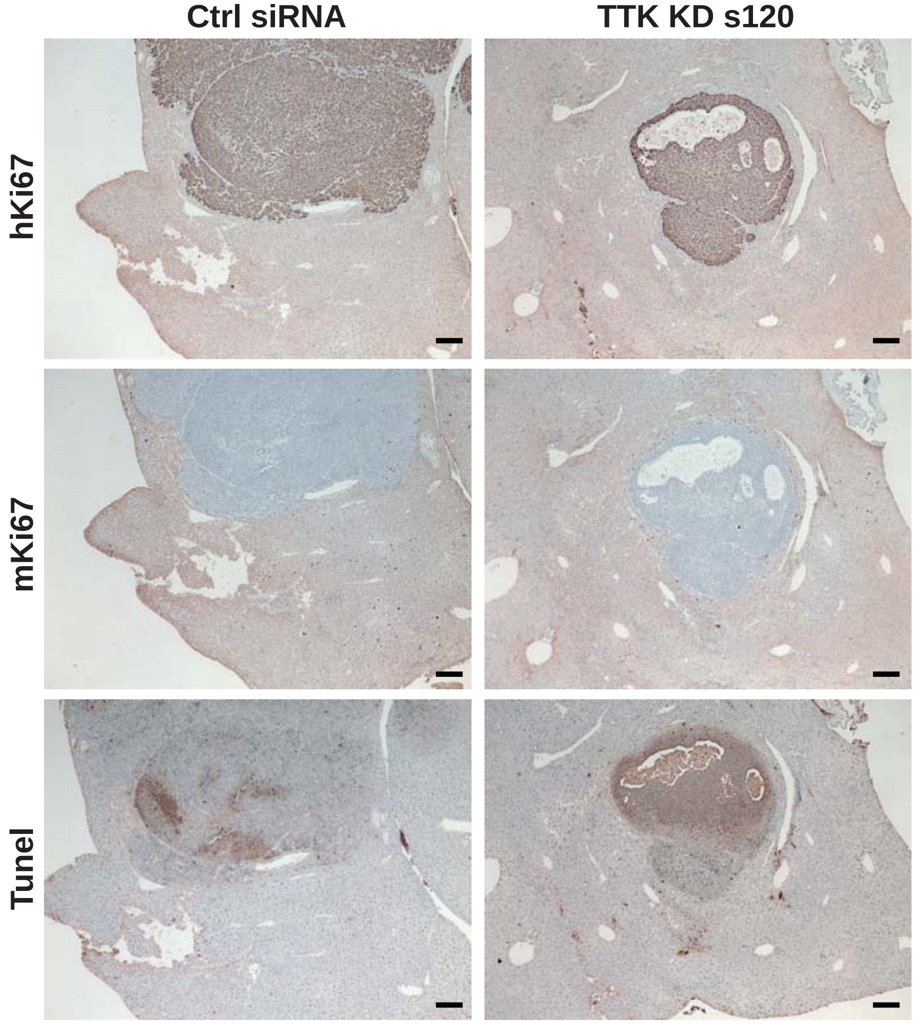

fig. S13

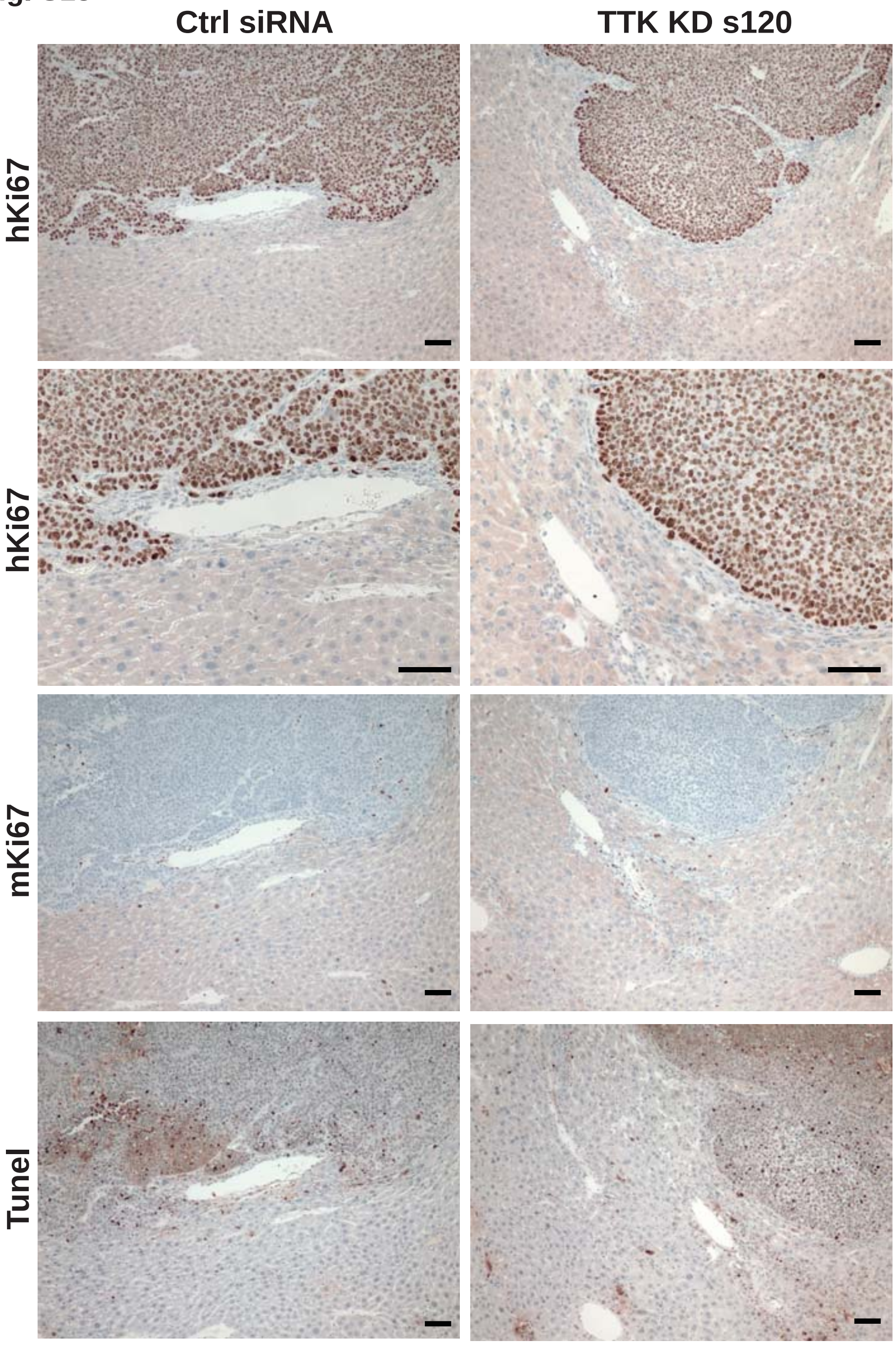

Supplement: Supplementary Information [file srep33121-s1.pdf]
